# Development and Psychometric Testing of the Breastfeeding Knowledge Scale (BKS) in Brazilian Healthcare Students

**DOI:** 10.1111/jep.70409

**Published:** 2026-03-15

**Authors:** Patrícia Lima Pereira Peres, Rachele Simeon, Maria Helena do Nascimento Souza, Giovanni Galeoto, Thaís Emanuele da Conceição, Anna Berardi, Donatella Valente, Rosane Harter Griep

**Affiliations:** ^1^ Faculdade de Enfermagem Universidade do Estado do Rio de Janeiro Rio de Janeiro Brasil; ^2^ Department of Neuroscience, Rehabilitation, Ophthalmology, Genetics and Maternal Child Health (DINOGMI) University of Genoa Genoa Italy; ^3^ Escola de Enfermagem Anna Nery Universidade Federal do Rio de Janeiro Rio de Janeiro Brasil; ^4^ Department of Human Neurosciences Sapienza University of Rome Rome Italy; ^5^ IRCSS Neuromed Pozzilli Isernia Italy; ^6^ Instituto Oswaldo Cruz Fundação Oswaldo Cruz Rio de Janeiro Brasil

**Keywords:** breastfeeding, clinical practice, health professions education, knowledge, psychometrics, students, validation study

## Abstract

**Rationale:**

Breastfeeding is recognized as a cornerstone of maternal and child health, yet health professionals frequently report insufficient knowledge to provide effective support. Strengthening the evaluation of educational outcomes in health professions training is therefore essential to improve clinical practice and patient care. This study aimed to develop and validate the Breastfeeding Knowledge Scale (BKS), a tool to assess breastfeeding‐related knowledge among health professions students in Brazil.

**Methods:**

This study followed COSMIN guidelines. Concept elicitation was conducted through workshops with experts and students to identify relevant domains of breastfeeding knowledge. A pilot study, including cognitive interviews with students, evaluated the comprehensibility and comprehensiveness of items. Content validity was assessed with both students and a multidisciplinary panel of professionals using the content validity index (CVI) and content validity coefficient (CVC), complemented by qualitative feedback. Psychometric testing included exploratory factor analysis (EFA) to evaluate structural validity and guide item reduction, followed by assessment of internal consistency with Cronbach's *⍺*, calculated only after confirming the unidimensionality of each subscale.

**Results:**

The concept elicitation process yielded five thematic clusters that were reorganized into three broader domains: Biological aspects of lactation, Policy and Sociocultural Context and Clinical Management (including complementary feeding). After iterative refinement and EFA, the instrument was reduced to 40 items. The final version was administered to 143 students. Internal consistency was excellent for the overall scale (Cronbach's *⍺* = 0.910) and strong for each domain (biological = 0.810; policy and sociocultural context = 0.790; clinical management = 0.796). These results supported both structural validity and reliability of the BKS.

**Conclusions:**

The BKS is a valid and reliable instrument for evaluating breastfeeding‐related competencies in health professions students. By providing a comprehensive and psychometrically sound measure, it enables rigorous evaluation of training adequacy, supports curriculum improvement and ultimately contributes to strengthening clinical practice and breastfeeding outcomes.

## 1 Development and Validation of Breastfeeding Knowledge Scale (BKS)

Breastfeeding is fundamental for ensuring infants' and women's health and well‐being, providing optimal nutrition and essential antibodies that protect against various diseases [1]. It has been shown to reduce the risk of infections, chronic conditions and even maternal postpartum depression while fostering a strong emotional bond between women and child [2]. Given its wide‐reaching benefits, the World Health Organization (WHO) and the American Academy of Pediatrics (AAP) recommend exclusive breastfeeding for the first 6 months of life, followed by continued breastfeeding and complementary foods for at least 2 years [2]. In Brazil, although most women initiate breastfeeding, over half of the children stop receiving exclusive breastfeeding within the first month of life. While there has been a positive trend towards increased breastfeeding rates in the country, we remain far from achieving the WHO recommendation of exclusive breastfeeding for the first 6 months and continued breastfeeding until at least 2 years of age or beyond [3]. In low‐ and middle‐income countries (LMICs), the risk of mortality in the early months of life is six to 10 times higher for infants who are not breastfed compared to those who are [4].

Numerous studies underscore the crucial role that health professionals play in promoting, protecting and supporting breastfeeding [5]. Research has shown that healthcare workers with adequate breastfeeding knowledge and skills are more likely to encourage and support breastfeeding women, leading to improved breastfeeding outcomes [6]. Health professionals' quality of breastfeeding support significantly affects a woman's decision to initiate and continue breastfeeding [7]. Therefore, equipping health professions students with a solid understanding of breastfeeding is essential for ensuring they are prepared to support and educate women effectively throughout the breastfeeding process.

In recognition of this, several international guidelines, including the *Core Competencies in Breastfeeding Care and Services for All Health Professionals* [8], emphasize that all health professionals, regardless of their specialization, should possess foundational knowledge of breastfeeding. These competencies include understanding the physiology of lactation, counselling techniques and strategies to overcome common breastfeeding challenges. Moreover, the WHO further highlights the need for healthcare workers to be proficient in breastfeeding support to achieve public health goals related to maternal and child health [9]. Although these international frameworks clearly define the competencies required for effective breastfeeding support, they provide limited guidance on how such competencies should be systematically assessed within health professions education. As a result, current training programmes often lack comprehensive and validated tools to evaluate whether students have acquired the full range of recommended breastfeeding competencies [10, 11].

Despite widespread recognition of breastfeeding's importance, research and education in the health professions continue to focus primarily on biological and clinical aspects, such as lactation physiology, breast anatomy and breastfeeding techniques [3]. Breastfeeding, however, is a complex practice influenced by factors that extend beyond biology, including sociocultural norms, legal protections and supportive policies. Sociocultural beliefs, misconceptions and stigma can substantially affect a woman's decision to initiate and sustain breastfeeding [12, 13]. Likewise, knowledge of breastfeeding‐related laws and policies, such as paid maternity leave and workplace accommodations, is associated with higher breastfeeding initiation and longer duration [7, 13]. Despite their relevance, these dimensions remain underrepresented in health professional training and are rarely assessed. As a result, inadequate preparation may lead to ineffective counselling, inconsistent advice and delayed management of breastfeeding difficulties, contributing to maternal distress and early cessation of breastfeeding [14]. Pre‐service education in the health professions is heterogeneous and largely non‐standardized, with wide variability in the content, duration and depth of breastfeeding education across undergraduate programmes in medicine, nursing and allied health disciplines [11]. Training is often fragmented and inconsistently integrated into curricula. Empirical studies consistently report insufficient breastfeeding knowledge among students, along with low self‐efficacy and limited confidence in counselling breastfeeding women and managing common breastfeeding challenges [15]. Moreover, the lack of comprehensive and validated assessment tools limits the systematic evaluation of breastfeeding‐related competencies and, consequently, hampers the identification of educational gaps [10]. This gap is particularly critical in Brazil, where structural constraints such as early return to work and lack of maternity leave increase the likelihood of exclusive breastfeeding discontinuation. Evidence from Brazilian health services also indicates gaps in breastfeeding‐related knowledge and practices among healthcare providers, suggesting shortcomings in pre‐service training and continuing education and highlights the need to qualify counselling, management and information delivered to mothers, especially in vulnerable groups [16]. Several instruments have been developed to assess breastfeeding knowledge [10]; however, most are designed for breastfeeding women or practising professionals and focus predominantly on biological and technical aspects of lactation. Few tools target health professions students, and those available often lack comprehensive coverage of sociocultural, legal and policy‐related competencies. In addition, existing instruments rarely incorporate items that capture context‐specific sociocultural and structural determinants of breastfeeding, particularly in low‐ and middle‐income settings. These limitations restrict their usefulness for evaluating pre‐service education and highlight the need for a psychometrically robust, context‐appropriate assessment tool.

Therefore, this study aims to develop and validate a comprehensive assessment tool, the BKS, to evaluate the breastfeeding‐related competencies of health professions students in Brazil. This tool is designed to provide a holistic assessment, covering not only biological knowledge but also the sociocultural, legislative and policy‐related aspects crucial for effective breastfeeding support.

## 2 Methods

**Figure 1:**
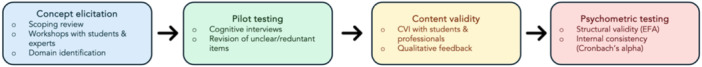
Flowchart of the development and validation process.

The steps of the process of validation of the Scala (Figure 1, Flowchart of the construction and validation process).

### 2.1 Identification of Existing Instruments

To identify existing assessment tools related to breastfeeding knowledge, a systematic literature search was conducted in PubMed, CINAHL, Embase, Scopus and PsycINFO. The following search string was used and adapted as appropriate for each database: (‘breastfeeding’ OR ‘breast‐feed’ OR ‘lactation’) AND (‘knowledge’ OR ‘awareness’) AND (‘students’ OR ‘healthcare students’ OR ‘medical students’ OR ‘nursing students’ OR ‘university students’) AND (‘Brazil’ OR ‘Brazilian’) AND (‘assessment’ OR ‘scale’ OR ‘questionnaire’ OR ‘instrument’). The review process followed PRISMA 2020 guidelines. After duplicate removal, titles and abstracts were screened according to predefined inclusion and exclusion criteria. Studies were considered eligible if they reported the development, validation or use of instruments assessing breastfeeding knowledge among students in Brazil. Full texts of potentially relevant articles were independently assessed for eligibility. For each identified instrument, psychometric properties were extracted, including reliability, validity and internal consistency. Methodological quality was appraised, when applicable, using the COSMIN (COnsensus‐based Standards for the selection of health Measurement INstruments) checklist [17].

### 2.2 Concept Elicitation

The BKS was developed to comprehensively assess knowledge of breastfeeding among health professions students in Brazil. To identify relevant concepts and generate items, three structured concept elicitation workshops were organized with a multidisciplinary group of participants. The first group consisted of five breastfeeding experts (paediatrician, nutritionist, nurse, physiotherapist, dentist). The second group included 35 health professions students (medicine, nursing, nutrition, dentistry, occupational therapy) from all years of study. Students were selected using a strategic non‐random sampling procedure designed to maximize variability in terms of degree programme and year of study. Although this approach may introduce selection bias, it was intentionally adopted to ensure broad conceptual representation rather than statistical generalizability. To mitigate potential bias, participants were recruited across multiple academic years and disciplines, and thematic patterns were compared across subgroups during analysis to identify convergence and divergence of perspectives. Recruitment continued until thematic redundancy was observed across workshops, suggesting adequate conceptual coverage. The third workshop was a mixed session with both experts and students to ensure integration of perspectives.

At the start of each workshop, participants were presented with the seeding statement: ‘Thinking as broadly as possible, please generate statements that describe what a health professions student should know in order to effectively support breastfeeding women’. Responses were written on individual cards and then intuitively sorted by participants into thematic categories. Participants were also asked to rate each statement according to two dimensions: importance for practice and relevance for student training.

The sorting and rating data were analysed during the workshop using consensus‐based clustering techniques. Clusters were subsequently discussed in plenary, where participants were asked to comment on the meaningfulness of groupings and to propose overarching descriptors for each category.

A scoping review on breastfeeding education for health professions students, combined with insights from an internal expert group and preliminary student interviews, provided the initial material for a draft concept elicitation protocol. The protocol included both general domains (overall breastfeeding knowledge, attitudes towards breastfeeding, role of the healthcare professional) and specific domains (biological aspects of lactation, sociocultural influences, policy and legal frameworks, clinical management and complementary feeding). During the workshops, participants were first asked to describe the essential characteristics they associate with competent breastfeeding support. They were then invited to respond to open‐ended questions such as: ‘What aspects of breastfeeding knowledge are most relevant to your future professional role?’ and ‘Which barriers or facilitators influence a health professional's ability to support breastfeeding women?’ Specific prompts followed, asking about how cultural beliefs, hospital practices or legislative protections affect breastfeeding outcomes. Participants also reflected on how breastfeeding education is currently addressed in their curricula and suggested additional content to be prioritized for training. Their answers were systematically collected and integrated into a structured script, which was piloted with one group of experts and one group of students to ensure clarity and comprehensiveness.

All focus group discussions and individual responses were audio‐recorded, transcribed verbatim and entered into qualitative analysis software. Three members of the research team independently reviewed the transcripts to identify recurring patterns in breastfeeding knowledge domains and developed preliminary coding frameworks. Intercoder agreement was assessed through independent double‐coding of a subset of transcripts, followed by comparison and discussion of coding discrepancies. Disagreements were resolved through iterative discussion until consensus was achieved, and the coding framework was subsequently refined and applied to the remaining transcripts. Data collection and analysis proceeded concurrently, and thematic saturation was considered achieved when no new codes or conceptual domains emerged across successive workshops.

### 2.3 Pilot Testing

To evaluate the comprehensibility and comprehensiveness of the BKS, a pilot study was conducted with health professions students representing the target population. Thirty students from medicine, nursing, nutrition and dentistry were recruited using purposive sampling through university mailing lists and classroom announcements to ensure representation across degree programmes and academic years [18]. The sample size was considered adequate for cognitive interviewing, as methodological guidance suggests that 15–30 participants are sufficient to identify most comprehension problems in questionnaire development [19].

Participants were invited to complete the BKS while taking part in cognitive interviews. They were first asked to comment on the instructions (clarity of wording, appropriateness for students), then on each item (relevance, precision and whether the meaning was clear), on the response options (completeness, appropriateness of scale points) and on the recall period (suitability for student knowledge). Open‐ended probes were used, such as: ‘Can you explain what you think this question is asking?’ and ‘Was there any part that was confusing or difficult to answer?’. All interviews were audio‐recorded, transcribed verbatim and reviewed by three independent researchers. Each analyst developed codes to classify problems in comprehension (e.g., unclear terminology, redundancy, inconsistent response scales). Using content analysis, these codes were organized into categories and compared until consensus was reached.

For each item or instruction flagged as problematic, a decision rule was applied: (1) items misunderstood by ≥ 20% of participants were rephrased; (2) items considered redundant were merged or removed; (3) items identified as incomplete were revised with additional content drawn from student feedback. This iterative refinement process addressed all problems before finalizing the instrument. Because the BKS is multidimensional (biological, sociocultural/policy and management subscales), problems regarding comprehensibility were examined separately per domain. This allowed us to document whether specific subscales posed more challenges and to resolve issues accordingly.

### 2.4 Content Validity

To further establish the content validity of the BKS, structured evaluations were conducted with both the target population (health professions students) and a multidisciplinary panel of healthcare professionals who were not involved in the earlier concept elicitation or pilot phases. Participants in both groups were asked to assess the relevance, comprehensiveness and comprehensibility of the scale. Each item was rated on a 5‐point Likert scale (1 = *strongly disagree*, 5 = *strongly agree*) for relevance.

The content validity index (CVI) was calculated at the item level (I‐CVI) as the proportion of raters assigning a score of 4 or 5 for each item. No scale‐level CVI was computed. Items with an I‐CVI < 0.80 were revised or removed. The content validity coefficient (CVC) was also calculated at the item level to adjust for potential rater bias and to provide a complementary estimate of agreement.

Both students and professionals were also invited to provide open‐ended feedback on whether the scale covered all relevant aspects of breastfeeding knowledge (comprehensiveness) and whether the wording, instructions, response options and recall periods were clear and understandable (comprehensibility). Qualitative feedback was systematically coded and synthesized by the research team into thematic categories. This dual evaluation ensured that the BKS captured the educational needs of students while remaining clinically meaningful and acceptable to practising professionals.

### 2.5 Psychometric Testing

Structural validity of the BKS was evaluated through exploratory factor analysis (EFA), as recommended in early‐stage instrument development when the underlying dimensional structure has not yet been empirically established. The final 40‐item version was administered to 143 health professions students. Sample size adequacy was evaluated considering commonly cited recommendations for factor analysis, which suggest a minimum of 100 participants and between four and 10 respondents per item [20]. Although the participant‐to‐item ratio was slightly below the upper recommended thresholds, the sample exceeded the minimum absolute sample size, and communalities were inspected to ensure factor stability. EFA with principal axis factoring and oblique rotation was performed on a randomly split half of the dataset to explore the underlying factor structure. Factor retention was based on eigenvalues > 1, scree plot inspection and parallel analysis. Given the exploratory nature of this initial validation study, confirmatory factor analysis (CFA) was not performed at this stage; independent replication and CFA are planned for future studies with larger samples, in line with COSMIN recommendations [21].

Internal consistency was examined for each identified factor, but only after unidimensionality was confirmed through the exploratory factor analyses. For each unidimensional subscale, Cronbach's *⍺* were calculated. Alpha and omega values ≥ 0.70 were considered acceptable, while values ≥ 0.90 indicated excellent internal consistency without redundancy.

Statistical analyses were performed using IBM‐SPSS version 23.00 software.

## 3 Results

### 3.1 Identification of Existing Instruments

The database search yielded a total of 43 records: seven from PubMed, 12 from CINAHL, 15 from Embase, eight from Scopus and one from PsycINFO. After removal of 29 duplicates, 14 records were screened by title and abstract, and four full‐text articles were assessed for eligibility. No studies met the predefined inclusion criteria (Supporting Information).

### 3.2 Concept Elicitation

The concept elicitation process generated a wide range of statements from both students and experts, which were organized into five main thematic clusters: biological aspects of lactation, sociocultural influences, policies and legal frameworks, clinical management and complementary feeding. Within these clusters, participants identified categories such as milk production and physiology, benefits of exclusive breastfeeding, cultural beliefs about feeding practices, maternity leave policies, hospital routines influencing breastfeeding, breastfeeding techniques and introduction of complementary foods. These categories reflected the breadth of knowledge expected of health professions students, encompassing both biomedical and contextual determinants of breastfeeding practices.

The clustering exercise, followed by consensus discussions in plenary sessions, confirmed that the categories were both mutually exclusive (concepts were clearly distinguishable from one another) and substantively exhaustive (covering the full range of ideas expressed). This indicated that concept saturation had been achieved, as no new domains emerged during the final sessions.

For questionnaire development, the five clusters were reorganized into three broader operational domains to enhance usability of the instrument: (i) Section 1: Biological aspects of breastfeeding (38 items), derived from the cluster on physiology and health outcomes. (ii) Section 2: Policy and sociocultural context (34 items), which integrated categories from the sociocultural influences cluster together with policy and legal frameworks. (iii) Section 3: Clinical management, including complementary feeding (28 items), which combined the cluster on clinical management with content on introduction of complementary foods.

This reorganization facilitated alignment between the conceptual framework and the practical structure of the instrument. The process yielded an initial pool of 100 items comprehensively addressing the construct of breastfeeding knowledge in health professions students.

### 3.3 Pilot Testing

The preliminary 100‐item version was pilot tested with 30 students from four health degree programmes. Cognitive interviews identified issues with 12 items: seven items were rephrased due to unclear terminology (e.g., use of technical jargon unfamiliar to early‐year students), three were merged to address redundancy between similar questions (overlapping items on breast milk storage) and two were revised to improve response option clarity. No item was judged irrelevant, and no major structural changes were required. Problems were distributed across domains, with slightly more revisions needed in the clinical management subscale. The decision rules defined a priori were consistently applied, and feedback was systematically organized into mutually exclusive and substantively exhaustive categories of issues. Pilot testing confirmed that instructions were clear, the recall period was appropriate and response options were well understood. Overall, revisions resulted in a reduction of three items, leading to a 97‐item version for subsequent psychometric evaluation. The final version retained the three‐domain structure, demonstrating saturation and confirming comprehensive coverage of breastfeeding knowledge.

### 3.4 Content Validity

Content validity was assessed with 30 health professions students and a multidisciplinary panel of 35 experts (paediatrics, nursing, nutrition, dentistry, physiotherapy, audiology). Each item's CVI and CVC were calculated. As shown in Table 1, nine items were removed due to CVI values < 0.80, and six items were rephrased based on lower CVC scores indicating variability among raters. Open‐ended feedback from both groups highlighted issues of wording clarity and redundancy in some items, particularly within the policy/sociocultural domain. No major gaps were identified, and participants confirmed that the scale comprehensively covered the domains of breastfeeding knowledge. Instructions and response options were judged clear and appropriate. These findings confirm that the final version of the BKS demonstrated strong content validity, balancing the educational needs of students with clinical applicability identified by professionals.

**Table 1:** Content validity index of the breastfeeding knowledge scale (BKS).

Item	CVC	CVI	Item	CVC	CVI
1	0.97	0.99	51	0.94	0.93
2	1	1	52	0.92	0.89
3	0.77	0.69*	53	0.92	0.92
4	0.99	1	54	0.96	0.94
5	0.81	0.78*	55	0.98	0.99
6	0.95	0.95	56	0.98	0.98
7	0.99	1	57	0.95	0.93
8	0.96	0.95	58	0.74	0.67*
9	0.91	0.89	59	0.97	0.96
10	0.99	1	60	0.94	0.93
11	0.97	0.97	61	0.98	0.98
12	0.75	0.69*	62	1	1
13	0.93	0.91	63	0.91	0.89
14	0.96	0.95	64	0.98	0.99
15	0.72	0.77*	65	0.94	0.93
16	1	1	66	0.95	0.95
17	0.98	0.99	67	0.97	0.96
18	0.98	0.99	68	0.98	0.98
19	0.94	0.93	69	0.98	0.98
20	1	1	70	0.95	0.93
21	0.92	0.91	71	0.94	0.92
22	0.91	0.9	72	0.97	0.96
23	0.91	0.89	73	0.92	0.88
24	0.81	0.78*	74	0.79	0.70*
25	0.98	0.98	75	0.95	0.93
26	0.95	0.95	76	0.95	0.93
27	0.98	0.99	77	0.99	1
28	0.97	0.96	78	0.92	0.92
29	0.99	1	79	0.97	0.96
30	0.92	0.91	80	0.95	0.93
31	0.95	0.95	81	0.99	1
32	0.98	0.99	82	0.97	0.97
33	0.99	1	83	0.91	0.9
34	0.9	0.88	84	0.94	0.92
35	0.97	0.96	85	0.97	0.97
36	0.95	0.95	86	0.81	0.76*
37	0.97	0.96	87	0.98	0.98
38	0.94	0.93	88	0.99	0.98
39	0.95	0.95	89	0.99	1
40	0.97	0.97	90	0.99	1
41	0.96	0.95	91	0.96	0.95
42	0.99	1	92	0.99	1
43	0.98	0.99	93	1	1
44	0.92	0.91	94	0.99	1
45	0.9	0.87	95	0.96	0.95
46	0.97	0.96	96	0.97	0.96
47	0.99	0.98	97	1	1
48	0.94	0.92	98	1	1
49	0.92	0.92	99	0.82	0.79*
50	0.97	0.96	100	0.99	0.98

Abbreviations: CVC, content validity coefficient; CVI, content validity index.

* *p* > 0.05.

### 3.5 Psychometric Testing

The final 40‐item version of the BKS was administered to a sample of 143 Brazilian students (Table 2). EFA supported a three‐factor structure corresponding to the hypothesized domains: Section 1: Biological aspects of breastfeeding, Section 2: Policy and sociocultural context and Section 3: Clinical management. Within each section, the EFA indicated sufficient unidimensionality, allowing subsequent evaluation of internal consistency.

**Table 2:** Demographic data of the participants for the psychometric testing.

Characteristics	Number (%)
Gender	
Female	123 (86.6)
Age mean (SD)	22.8 (0.3)
Graduation programme	
Nursing	109 (76.8)
Medicine	21 (14.8)
Nutrition	8 (5.6)
Speech‐language therapy	2 (1.4)
Dentistry	1 (0.7)
Occupational therapy	1 (0.7)
Year	
First	36 (25.3)
Second	82 (57.7)
Third	24 (17)

Cronbach's ⍺ coefficients demonstrated good reliability: 0.810 for Section 1 (Biological), 0.790 for Section 2 (Policy and sociocultural context) and 0.796 for Section 3 (Management). The Biological domain included items assessing core physiological knowledge, such as mechanisms of milk production and hormonal regulation of lactation. The Policy and Sociocultural Context domain comprised items addressing maternity leave legislation, cultural beliefs influencing breastfeeding practices and hospital policies related to breastfeeding support. The Clinical Management domain included items focused on practical competencies, such as identification and management of common breastfeeding difficulties (e.g., mastitis, latch problems) and counselling strategies for breastfeeding mothers. The overall scale showed excellent internal consistency with *α* = 0.910 (Table 3). These findings indicate that the BKS is internally coherent within each domain and across the full instrument.

**Table 3:** Internal consistency of the breastfeeding knowledge scale (BKS).

	Cronbach's *⍺* if an item was deleted	
3	Ter uma norma escrita quanto às ações de promoção, proteção e apoio ao aleitamento materno é um dos 10 passos da Iniciativa Hospital Amigo da Criança.	0.905
6	A alimentação é um fator de identidade de povos e grupos e diz respeito aos afetos, ao cuidado consigo mesmo, com os outros e com o meio ambiente.	0.905
8	A amamentação está associada a efeitos benéficos na vida adulta.	0.906
10	A assistência à amamentação exige do profissional de saúde uma relação dialógica entre os saberes científicos e populares.	0.906
11	A composição nutricional do leite muda ao longo de uma mamada.	0.906
20	A mastite não é contraindicação expressa à amamentação.	0.907
22	A Organização Mundial da Saúde (OMS) e o Ministério da Saúde (MS) recomendam o aleitamento materno até os dois anos ou mais, sendo exclusivo nos primeiros seis meses de idade do bebê.	0.905
28	A rede de apoio social da mãe que amamenta interfere no êxito da prática da amamentação	0.905
32	A sucção da mama pelo bebê é o principal estímulo para a produção do leite materno.	0.905
35	Alojamento conjunto é um sistema hospitalar em que o recém‐nascido sadio, logo após o nascimento, permanece 24 horas por dia ao lado da mãe até a alta hospitalar.	0.904
43	As unidades básicas de saúde devem estar estruturadas para praticar o acolhimento e a assistência à mulher, ao bebê e à família.	0.907
46	Bebês prematuros que recebem leite materno têm menor risco de desenvolver enterocolite necrosante devido aos anticorpos IgA do leite humano.	0.906
47	Coletar, processar e distribuir leite humano a bebês prematuros e de baixo peso internados são alguns dos objetivos da Rede Global de Bancos de Leite (rBLH).	0.904
48	Desde o primeiro mês, deve‐se iniciar a higiene oral do bebê com uso de escova macia e pasta de dente para prevenção da cárie.	0.906
50	Disfunções orais no bebê estão associadas às dificuldades na sucção, baixo ganho de peso e traumas mamilares.	0.906
52	É proibida a promoção comercial de fórmulas lácteas para lactentes menores de 12 meses, bicos, mamadeiras, chupetas e protetores de mamilos.	0.906
54	Em geral, o conselho mais adequado para dar a uma mulher com baixa produção de leite é aumentar a ingesta de líquidos.	0.906
56	Independente da renda familiar o leite materno é mais benéfico do que a fórmula láctea.	0.906
57	Ingurgitamento mamário é uma estase láctea decorrente do não esvaziamento da mama ou mamadas infrequentes.	0.904
59	Mulheres com antecedentes de ansiedade e depressão não devem amamentar.	0.904
61	No caso de a aréola estar tensa, sugere‐se leve massagem na região mamilo‐areolar, extrair manualmente um pouco de leite para a aréola ficar mais macia e facilitar a pega.	0.905
63	Numa pega adequada, a língua vem à frente, sobre a gengiva inferior; os lábios ficam virados para fora; as bochechas ficam arredondadas e o queixo do bebê fica encostado na mama.	0.904
64	O álcool ingerido pela mãe é transferido para o leite materno onde pode permanecer por até duas horas.	0.906
65	O aleitamento materno não está contemplado na Política Nacional de Atenção à Saúde da Criança.	0.906
66	O contato pele a pele no pós‐parto imediato contribui para o êxito da amamentação	0.904
69	O leite materno é capaz de suprir todas as necessidades nutricionais do bebê até os 6 meses de vida, e mesmo em países de clima quente não é necessário oferecer água ao bebê.	0.904
70	O leite materno ordenhado deve ser ofertado para o bebê preferencialmente por meio de um copinho.	0.904
72	O Método Canguru constitui uma política pública fundamentada no contato pele a pele entre mãe e o recém‐nascido de baixo peso e/ou prematuro.	0.904
74	O uso da mamadeira está associado à maior incidência de má oclusão dentária, cárie precoce da infância, otite média e de candidíase oral.	0.904
75	O uso de antibiótico é contraindicação expressa para a amamentação.	0.904
76	O uso de intermediários pode trazer prejuízos à amamentação, devendo ser evitado.	0.905
77	O volume de leite materno ingerido a cada mamada é regulado pelo bebê.	0.904
81	Os profissionais de saúde não podem receber brindes, presentes e/ou vantagens financeiras das indústrias de leites, fórmulas infantis, chupetas, mamadeiras e protetores de mamilos.	0.906
82	Para amamentar seu filho, inclusive se advindo de adoção, até que este complete 6 (seis) meses de idade, a trabalhadora formal (CLT) terá direito, durante a jornada de trabalho, a 2 (dois) descansos especiais de meia hora cada um.	0.906
83	Para aumentar a produção de leite, a lactante deve consumir cerveja preta e canjica.	0.906
84	Para evitar a confusão de bico e outros problemas bucais a chupeta deve ser evitada.	0.904
85	Para que o método da amenorreia lactacional seja efetivo são necessárias: ausência de menstruação, amamentação exclusiva sob livre demanda e que o bebê tenha menos de 6 meses de vida.	0.906
86	Para um bebê que continua chorando após a mamada recomenda‐se complementar com fórmula láctea.	0.905
88	São sinais de fome no bebê: inquietação aparente; vira o rosto para os lados, abrindo a boca; suga as mãos e o choro.	0.905
89	Tubérculos de Montgomery são glândulas que armazenam o leite na mama.	0.906
Cronbach's *⍺* total		0.907

## 4 Discussion

The primary objective of this study was to develop and validate a multidimensional assessment scale designed to evaluate breastfeeding‐related competencies among health professions students in Brazil. This instrument, known as the BKS, aims to provide a comprehensive evaluation that encompasses not only biological knowledge and the clinical management of socio‐cultural, legislative and policy‐related dimensions essential for delivering holistic breastfeeding support. By integrating these dimensions, the BKS addresses the multifactorial nature of breastfeeding support. The questionnaire was developed through a structured multi‐phase process including literature review, qualitative input and expert consensus.

Although several instruments assessing breastfeeding knowledge have been developed in different contexts, many focus primarily on biological aspects of lactation or on clinical management skills, with limited integration of sociocultural, legislative and policy‐related dimensions. In addition, some existing tools were designed for practising healthcare professionals rather than students, or lack comprehensive psychometric validation. In contrast, the BKS was specifically developed for health professions students and adopts a multidimensional framework that reflects the complexity of breastfeeding support within contemporary healthcare systems. By integrating biological knowledge, clinical management and sociopolitical context within a single validated structure, the BKS advances existing approaches by offering a more holistic and educationally oriented assessment tool.

A high level of consensus was achieved across Delphi rounds, supporting the content validity of the instrument. Notably, a consensus rate of 100% in the second round indicates the validity and quality of the questionnaire [22]. The panel comprised 35 experts, aligning with the recommendation of Steiner and Norman, who suggest a panel size of 3–10 experts to ensure a balanced and informed evaluation [23]. The anonymity of the Delphi process further minimized the risk of experts being influenced by one another's opinions. The questionnaire's metrics for content validity and internal consistency surpassed acceptable thresholds, reinforcing the instrument's methodological rigour [24]. The CVI exceeded 0.8 for each section, confirming that the included items effectively addressed our objectives and sufficiently represented the content domain [25].

The pilot study demonstrated a high response rate, suggesting that the questionnaire's length did not hinder completion. Although the average completion time remains undocumented, previous research indicates that questionnaire length does not significantly impact response quality [26]. Clarifying the language used in the question formulation probably facilitated participant understanding, aligning with the researchers' expectations for informative content.

Reliability analyses yielded substantial Cronbach's *⍺* values exceeding 0.7, supporting the coherence of the identified domains. An increased sample size in future studies is anticipated to yield even higher Cronbach's *⍺* scores [21]. Nevertheless, these substantial reliability values support the questionnaire's capacity to test potential associations between knowledge, attitudes and practices in breastfeeding [27].

During the item generation phase, significant attention was dedicated to crafting questions that were specific, clear and free from ambiguity. Each item focused on a single concept while avoiding negative wording. The language and reading levels were tailored to align with the target population's general education and health literacy levels, as recommended in the literature [28]. Furthermore, item redundancy was strategically included to ensure comprehensive coverage of the construct's various aspects during the early stages of scale development [23].

Adequate breastfeeding knowledge among healthcare professionals is essential to promote and sustain breastfeeding practices. These practitioners are crucial in promoting, supporting and facilitating breastfeeding among women. By equipping future health professionals with a robust understanding of breastfeeding—including its biological underpinnings, socio‐cultural contexts and relevant legislative frameworks—we can cultivate an environment that encourages and normalizes breastfeeding practices [29].

The overall Cronbach's *⍺* of 0.910 indicates excellent internal consistency. However, given the multidimensional structure of the BKS and the deliberate inclusion of conceptually related items to ensure comprehensive coverage of breastfeeding knowledge domains, the observed *⍺* is likely reflective of coherent domain representation rather than excessive duplication. Future studies may explore item reduction strategies using CFA or item response theory models to further optimize scale efficiency while preserving content breadth.

An additional consideration concerns the Brazilian educational and sociocultural context in which the BKS was developed. Breastfeeding promotion in Brazil is strongly shaped by national public health policies, maternity protection legislation and institutional initiatives aimed at supporting breastfeeding practices. These contextual factors likely influenced both the relevance of specific items, particularly those addressing policy and sociocultural dimensions, and the emergence of a multidimensional structure integrating biological, clinical and legislative aspects [10, 11]. Furthermore, curricular organization within Brazilian health professions programmes may have shaped students' perceived competencies and knowledge domains. As a result, while the BKS reflects priorities and realities of the Brazilian healthcare and educational system, caution is warranted when applying the instrument in different cultural or regulatory contexts. Cross‐cultural adaptation and external validation studies are necessary to examine whether the identified domains remain stable and relevant in other countries or educational systems.

From an educational perspective, the multidimensional structure of the BKS has important implications for curriculum design in health professions programmes. The identification of three distinct domains, biological knowledge, policy and sociocultural context and clinical management, suggests that breastfeeding education should extend beyond physiological mechanisms to include legislative frameworks, institutional policies and culturally sensitive counselling competencies. The scale can therefore serve as a diagnostic tool to identify curricular gaps within specific domains and to guide targeted pedagogical interventions. For example, low scores in the policy and sociocultural domain may indicate the need to integrate content on maternity protection legislation, workplace breastfeeding rights or community‐based promotion strategies. Similarly, results in the clinical management domain can inform the incorporation of simulation‐based training or case‐based learning focused on common breastfeeding challenges.

Moreover, the BKS may be used to evaluate the effectiveness of educational programmes over time, supporting evidence‐based curriculum reform. At the policy level, aggregated data derived from the scale could inform institutional strategies aimed at strengthening breastfeeding education within undergraduate health training, aligning academic preparation with national public health priorities.

Despite the methodological rigour adopted throughout the development and validation process, several limitations should be acknowledged. First, structural validity was examined using EFA without subsequent CFA. Although EFA is appropriate in early‐stage instrument development, the absence of confirmatory validation limits the strength of inferences regarding the stability of the factor structure. Future studies with larger and independent samples should perform CFA to confirm the dimensional structure identified in this study. Second, additional measurement properties recommended by the COSMIN framework—such as test–retest reliability, measurement error and responsiveness—were not assessed. The lack of temporal stability and measurement precision indicators may limit conclusions about the instrument's reliability over time and its sensitivity to change, particularly in educational intervention contexts. Third, the sample used for psychometric testing presented limited heterogeneity, as participants were recruited from a single professional training context. This may restrict the generalizability of findings to other health professions programmes or educational settings. Broader multisite validation studies involving diverse student populations are warranted to strengthen external validity.

Together, these considerations indicate that while the BKS demonstrates promising preliminary psychometric properties, further validation work is necessary to consolidate its robustness and applicability across contexts.

## 5 Conclusions

In conclusion, fostering a comprehensive understanding of breastfeeding among health professions students is essential to strengthening the quality of maternal and child care. The BKS provides a structured and psychometrically supported instrument for assessing breastfeeding‐related knowledge across biological, clinical and sociocultural domains within the Brazilian educational context. In its current validated form, the BKS should be considered primarily as a tool for evaluating knowledge and identifying curricular strengths and gaps in undergraduate health training programmes.

While the scale offers a framework that may inform curriculum development and educational planning, its direct impact on breastfeeding initiation, continuation or maternal outcomes was not assessed in this study. Moreover, additional measurement properties—such as CFA, test–retest reliability and responsiveness—remain to be established to further strengthen the robustness of the instrument.

Future research should therefore focus on confirmatory validation in larger and more diverse samples, longitudinal evaluation of temporal stability and formal cross‐cultural adaptation before implementation in different educational or healthcare systems. Through continued validation and contextual adaptation, the BKS has the potential to contribute to evidence‐informed breastfeeding education both in Brazil and internationally.

## Conflicts of Interest

The authors declare no conflicts of interest.

## Data Availability

The data that support the findings of this study are available on request from the corresponding author. The data are not publicly available due to privacy or ethical restrictions.
